# Retraction: Oroxylin a Inhibits the Protection of Bone Marrow Microenvironment on CML Cells Through CXCL12/CXCR4/P-gp Signaling Pathway

**DOI:** 10.3389/fonc.2020.00714

**Published:** 2020-04-20

**Authors:** Frontiers Editorial Office

The journal retracts the 03 April 2019 article cited above.

Following publication, concerns were raised regarding the integrity of western blot images. An investigation was therefore conducted in accordance with our established procedures, revealing that image duplication had taken place. The authors failed to provide satisfactory raw data and requested to have this article retracted.

This retraction was approved by the Chief Editors of Frontiers in Oncology and the Editor-in-Chief of Frontiers. The authors concur with the retraction and sincerely regret any inconvenience this may have caused to the reviewers, editors, and readers of Frontiers in Oncology.

